# Identification of proteins regulated by chlorogenic acid in an ischemic animal model: a proteomic approach

**DOI:** 10.1186/s42826-023-00164-5

**Published:** 2023-06-05

**Authors:** Murad-Ali Shah, Ju-Bin Kang, Phil-Ok Koh

**Affiliations:** Department of Anatomy, College of Veterinary Medicine, Research Institute of Life Science, Gyeongsang National University, 501 Jinjudaero, Jinju, 52828 South Korea

**Keywords:** Chlorogenic acid, Middle cerebral artery occlusion, Neuroprotection, Stroke

## Abstract

**Background:**

Cerebral ischemia is a serious neurological disorder that can lead to high morbidity and mortality. Chlorogenic acid is a polyphenol compound with antioxidant that can regulate proteins in cerebral ischemia. Middle cerebral artery occlusion (MCAO) surgery was performed to induce ischemic brain injury and was maintained for 24 h. Chlorogenic acid (30 mg/kg) or vehicle was administrated into the peritoneal cavity 2 h after MCAO surgery. The cerebral cortical tissues were collected for further study and a proteomic approach was performed to identify the proteins changed by chlorogenic acid in the MCAO animals.

**Results:**

We found that chlorogenic acid alleviated in changes in adenosylhomocysteinase, glycerol-3-phosphate dehydrogenase, eukaryotic translation initiation factor 4A-II, apolipoprotein A-I, and mu-crystallin. These proteins were reduced in MCAO animals with vehicle, and these reductions were attenuated by chlorogenic acid treatment. The mitigation of this reduction by chlorogenic acid was confirmed by the reverse transcription PCR technique. These proteins are associated with energy metabolism, protein synthesis, inflammation, and physiological metabolism. They are involved in the neuroprotective effect of chlorogenic acid. These results showed that chlorogenic acid alleviates the neurological disorders caused by MCAO and regulates the expression of proteins involved in neuroprotection.

**Conclusions:**

Therefore, our findings provide evidence that chlorogenic acid plays a neuroprotective role in stroke animal models by controlling specific proteins.

**Supplementary Information:**

The online version contains supplementary material available at 10.1186/s42826-023-00164-5.

## Background

Stroke is the second leading cause of death worldwide, with 5.5 million deaths per year, and survivors are at high risk of chronic disorders [1]. Stroke is caused by a lack of blood supply and causes serious neurological disorders [2]. Stroke patients show signs of unilateral weakness, motor failure, speech impairment, numbness, and visual loss [3]. Ischemic and hemorrhagic strokes account for 80% and 20% of strokes, respectively [4]. In the case of ischemic stroke, low blood supply to the brain causes hypoxia and hypoglycemia, causing infarction of brain tissue [5]. Ischemic stroke causes serious brain edema and infarction, and histopathological changes including nuclear condensation and cytoplasmic expansion in neurons [6, 7]. It disrupts the blood brain barrier and increases inflammatory factors, cytokines, and chemokines, leading to inflammatory responses and pathological lesions [8, 9]. It also increases oxidative stress and cytoplasmic calcium concentrations, and finally induces necrosis and apoptosis of neuronal cells [8, 9].

Phenolic compounds have been recognized for their beneficial effects on anti-inflammatory and antioxidant properties [10]. Chlorogenic acid is a common phenolic compound that is present in coffee and tea [11]. It has anti-oxidative and anti-inflammatory effects and neuroprotective effects on ischemic neurological damage [12–14]. Chlorogenic acid also improves memory loss and prevents neuronal cell death by cerebral ischemia [11, 15]. Chlorogenic acid alleviates the production of reactive oxygen species and the increase of apoptotic proteins in ischemic brain injury [16, 17]. We previously reported that chlorogenic acid attenuates neurobehavioral impairment and reactive oxygen species production due to MCAO damage [18]. Chlorogenic acid acts as a potent neuroprotective agent by regulating apoptosis-associated proteins such as caspase-3, caspase-7 and poly ADP-ribose polymerase expression [18]. Chlorogenic acid attenuates the activity of microglia and astrocytes and regulates pro-inflammatory proteins against cerebral ischemic injury [19]. Chlorogenic acid also regulates the PI3K-Akt signaling pathway in cerebral ischemia, which activates phospho-Akt and phospho-Bad and alleviates the reduction of phospho-Bad and 14-3-3 binding during MCAO injury [20]. Our previous work demonstrated that chlorogenic acid modulates various mechanisms and pathways to protect neurons from ischemic injury. As mentioned above, chlorogenic acid has various advantages, but the mechanism of chlorogenic acid is very complex and ambiguous. We propose that chlorogenic acid exerts neuroprotective effects in cerebral ischemia and regulates several proteins that provide a neuroprotective function. Therefore, this study was performed to identify specific proteins regulated by chlorogenic acid in stroke animal models. We employed the proteomic technique to identify these proteins.

## Results

### Attenuation of oxidative stress by chlorogenic acid in MCAO damage

Our supplemental data showed that chlorogenic acid mitigates the increase in oxidative stress caused by MCAO damage. Reactive oxygen species (ROS) and lipid peroxidation (LPO) assays were performed to measure oxidative stress. 2′7′‐dichlorofluorescein (DCF) and malondialdehyde (MDA) levels as biomarkers of ROS and LPO were investigated. MCAO damage increased DCF and MDA levels, and chlorogenic acid treatment attenuated these increases. DCF levels were 1.82 ± 0.11 and 0.93 ± 0.09 in vehicle + MCAO animals and chlorogenic acid + MCAO animals, respectively (Additional file 1). MDA levels were 1.54 ± 0.10 in vehicle + MCAO animals and 0.89 ± 0.08 in chlorogenic acid + MCAO animals (Additional file 1). We confirmed the anti-oxidative effect of chlorogenic acid on cerebral ischemia.

### Identification of various proteins regulated by chlorogenic acid in MCAO damage

**Fig. 1:**
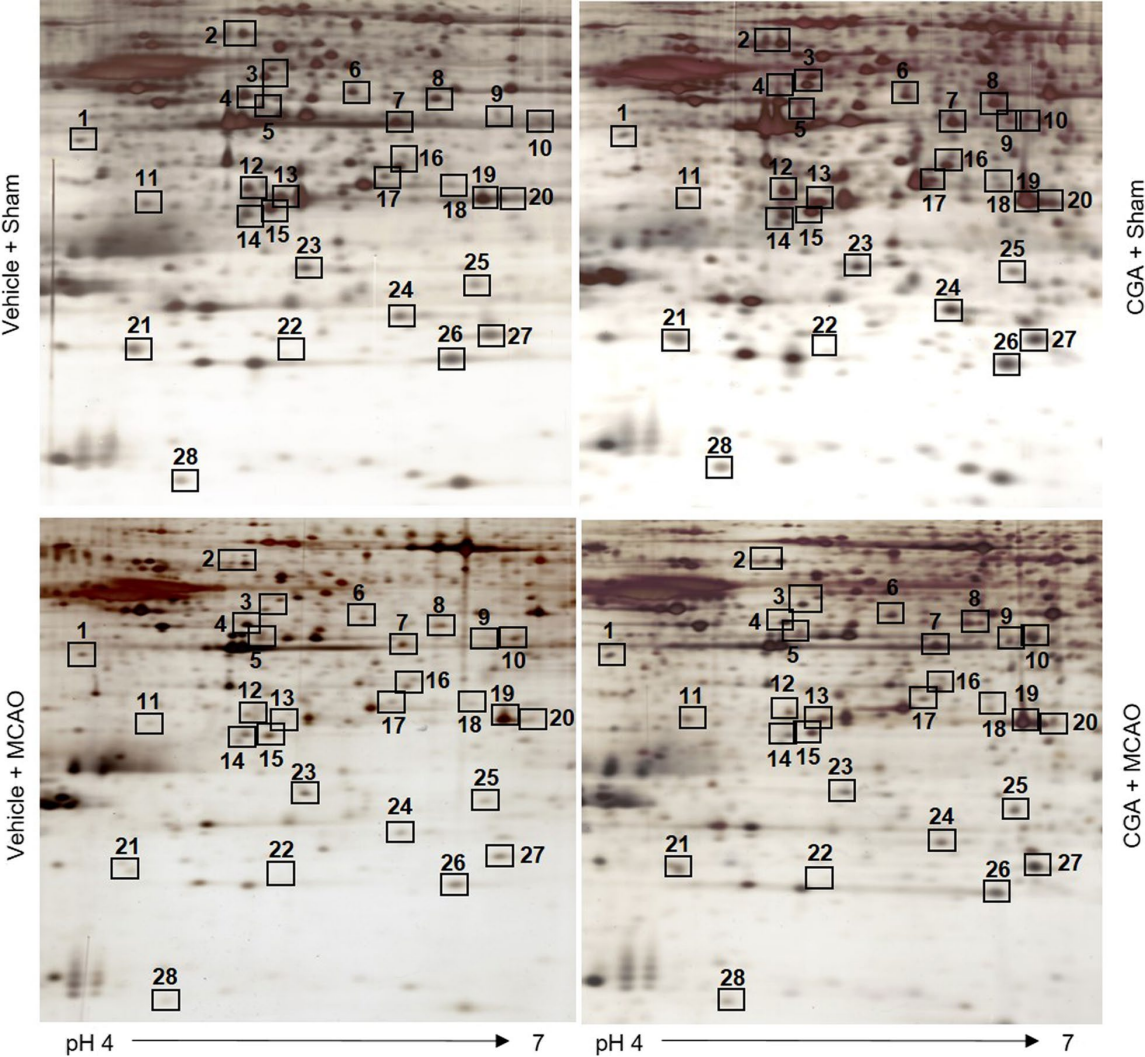
Chlorogenic acid regulates various proteins in the cerebral cortex during MCAO damage. Image of two-dimensional gel electrophoresis analysis in the cerebral cortex from vehicle + sham, chlorogenic acid (CGA) + sham, vehicle + middle cerebral artery occlusion (MCAO), CGA + MCAO animals. Isoelectric focusing was performed at pH 4–7 IPG strips and electrophoresed on 7.5–17.5% gradient SDS gels. Squares represent the identified protein spots

We identified proteins that were changed by chlorogenic acid in the cerebral cortex of the ischemic animal model using the proteomic technique. Two-dimensional gel image maps showed detected proteins in vehicle- or chlorogenic acid-treated animals with MCAO damage (Fig. 1). Almost 788 protein spots were observed in each gel image. There were 28 protein spots that exhibited a more than 2.5-fold intensity difference between vehicle- and chlorogenic acid-treated animals with MCAO damage. Among these detected protein spots, 25 spots were identified by MALDI-TOF analysis, and protein sequence coverage was 21–58% (Table 1). However, three protein spots did not match and were named as unknown.

**Table 1:** List of identified differentially expressed proteins in vehicle and chlorogenic acid-treated animals with middle cerebral artery occlusion

Spot no	Protein name	Accession no	Mw (kDa)	pI	Mass matched	Sequence coverage (%)
1	Gamma enolase	P07323	47.14	5.00	14/70	35
2	Unknown					
3	Rab, GTPase-GDP dissociation stimulation stimulator 1	P52306	66.32	5.20	20/156	53
4	Eukaryotic initiation factor 4A-II	Q5RKI1	46.37	5.33	16/82	40
5	Eukaryotic initiation factor 4A-II	Q5RKI1	46.37	5.33	16/82	40
6	Unknown	Q7TQ13	31.27	4.85	11/66	58
7	Rab GDP dissociation inhibitor beta	P50399	50.50	5.90	9/150	23
8	Rab GDP dissociation inhibitor beta	P50399	50.50	5.90	16/138	46
9	Protein phosphatase 2A, regulatory subunit B	P58389	36.59	5.88	9/56	29
10	Adenosylhomocysteinase	P10760	47.55	6.07	15/132	33
11	Thioredoxin	Q920J4	32.23	4.84	8/87	42
12	Mu-crystallin	Q9QYU4	33.53	5.34	9/86	24
13	Mu-crystallin	Q9QYU4	33.55	5.30	6/114	21
14	Mu-crystallin	Q9QYU4	33.53	5.34	9/86	24
15	Protein phosphatase 2A, subunit A	P63331	35.60	5.30	13/110	45
16	Unknown					
17	Isocitrate dehydrogenase (NAD+) subunit alpha	Q99NA5	39.59	6.47	7/93	31
18	Isocitrate dehydrogenase (NAD+) subunit alpha	Q99NA5	39.59	6.47	7/108	21
19	Isocitrate dehydrogenase (NAD+) subunit alpha	Q99NA5	39.59	6.47	7/93	31
20	Dynamin-like protein 1	Q8K1M6	80.24	6.99	14/90	22
21	Glycerol-3-phosphate dehydrogenase	O35077	37.43	6.16	8/105	25
22	Peroxiredoxin 2	Q61171	21.64	5.30	9/51	46
23	Stathmin	P16949	49.93	5.35	9/23	21
24	Apolipoprotein A-I	P04693	30.04	5.25	16/116	50
25	Alpha-synuclein	P37377	14.50	4.70	6/134	43
26	UMP-CMP kinase	Q4KM73	22.16	5.66	10/114	50
27	Proteasome subunit β 4	P34067	29.18	6.45	78/13	42
28	Proteasome subunit alpha type3	P18422	28.40	5.30	7/112	27

Protein names and accession numbers are listed according to the SWISS-PROT database

*MW* molecular weight, *pI* isoelectric point

### Regulation of specific proteins by chlorogenic acid in MCAO damage

**Fig. 2:**
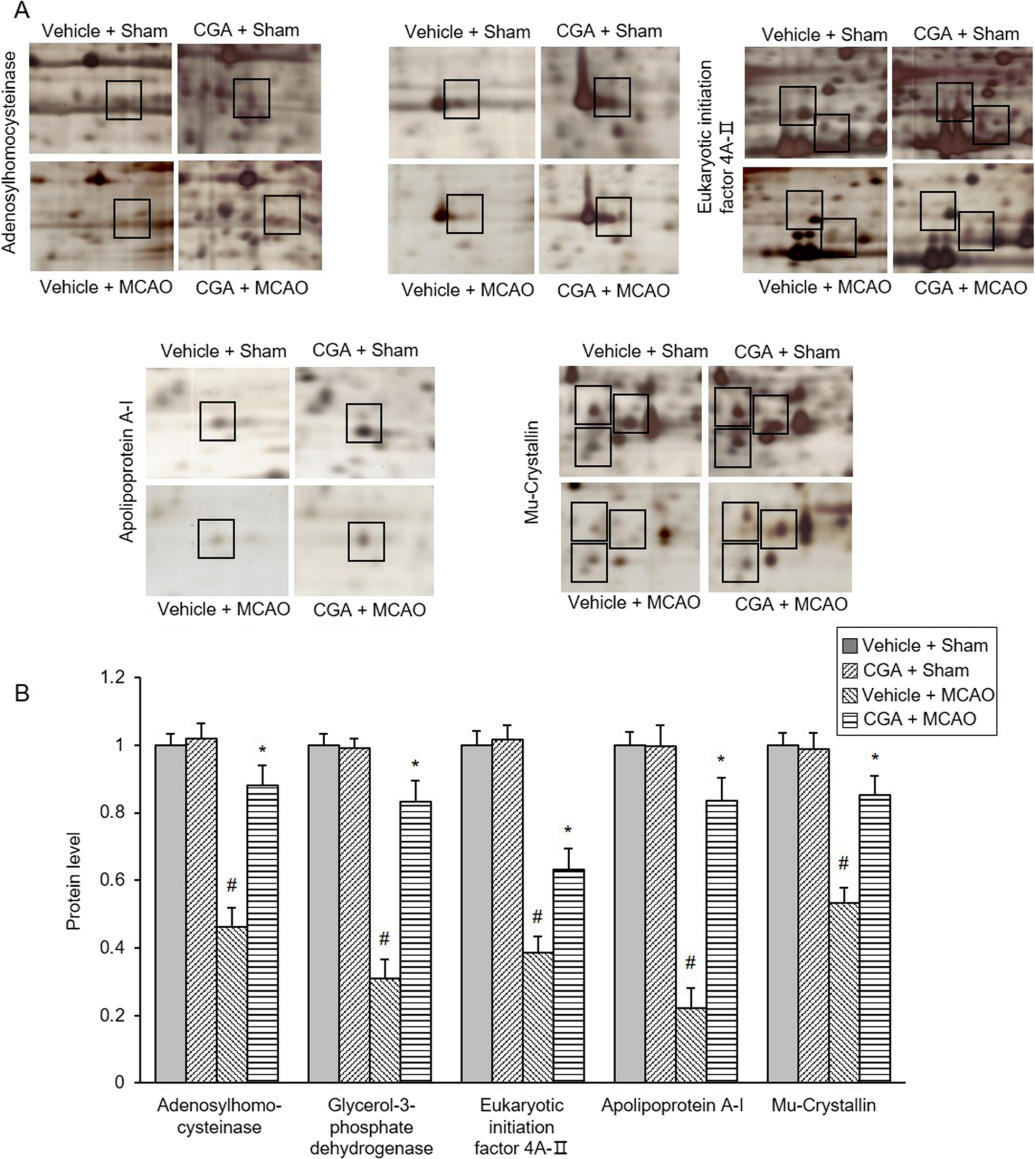
Chlorogenic acid alleviates changes in specific proteins due to MCAO damage. Magnified protein spots of adenosylhomocysteinase, glycerol-3-phosphate dehydrogenase, eukaryotic translation initiation factor 4A-II, apolipoprotein A-1, and mu-crystallin in the cerebral cortex from vehicle + sham, chlorogenic acid (CGA) + sham, vehicle + middle cerebral artery occlusion (MCAO), CGA + MCAO animals (**A**). Each square indicates the protein spots. The intensity of vehicle + sham animals was set to 1. The intensity of protein spot is represented as a ratio of that of vehicle + sham (**B**). Data (*n* = 3 per group) are represented as the mean ± S.E.M. #*p* < 0.01 versus vehicle + sham animals, *p < 0.05 versus vehicle + MCAO animals

We focused on the expression of adenosylhomocysteinase, eukaryotic initiation factor 4A-II, apolipoprotein A-I, mu-crystallin, and glycerol-3-phosphate dehydrogenase. We found that the spot intensity of these proteins was significantly decreased in the MCAO animals with vehicle compared to the sham animals. However, chlorogenic acid alleviated the decrease in these proteins (Fig. 2A). The spot density of these proteins was similar in sham animals regardless of treatment with vehicle or chlorogenic acid. The level of expression of this protein represents the ratio of the density of each group to the density of vehicle + sham group (Fig. 2B). Adenosylhomocysteinase levels were 0.46 ± 0.06 in vehicle + MCAO and 0.88 ± 0.05 in chlorogenic acid + MCAO. Glycerol-3-phosphate dehydrogenase levels were 0.31 ± 0.06 and 0.83 ± 0.06 in vehicle + MCAO and chlorogenic acid + MCAO animals, respectively. Eukaryotic initiation factor 4A-II levels were 0.39 ± 0.05 and 0.63 ± 0.06 in vehicle + MCAO and chlorogenic acid + MCAO animals, respectively. Apolipoprotein A-I levels were 0.22 ± 0.06 in vehicle + MCAO and 0.83 ± 0.07 in chlorogenic acid + MCAO animals. Mu-crystallin levels were 0.53 ± 0.04 in vehicle + MCAO and 0.85 ± 0.05 in chlorogenic acid + MCAO animals.

**Fig. 3:**
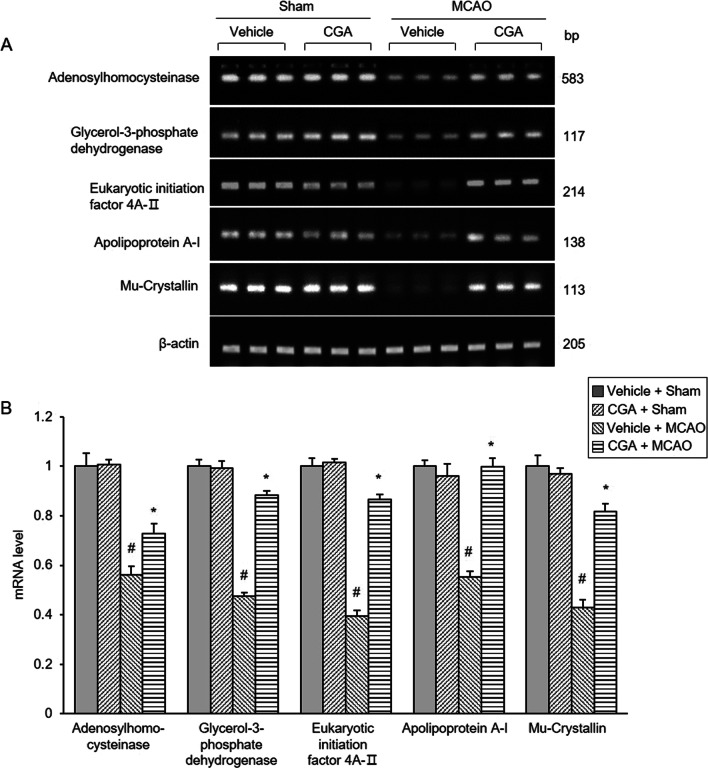
Chlorogenic acid alleviates the change of mRNA levels of specific proteins due to MCAO damage. Reverse transcription PCR products of adenosylhomocysteinase, glycerol-3-phosphate dehydrogenase, eukaryotic translation initiation factor 4A-II, apolipoprotein A-1, and Mu-crystallin in the cerebral cortex from vehicle + sham, chlorogenic acid (CGA) + sham, vehicle + middle cerebral artery occlusion (MCAO), CGA + MCAO animals (**A**). The band intensity of PCR product is represented as a ratio of β-actin product intensity, and the levels of vehicle + sham was set to 1 (**B**). Data (*n* = 5 per group) are represented as the mean ± S.E.M. #*p* < 0.05 versus vehicle + sham animals, *p < 0.01 versus vehicle + MCAO animals

We also confirmed these changes using reverse transcription PCR analysis. The expression of these mRNA was significantly decreased in MCAO animals with vehicle compared to sham animals, and chlorogenic acid attenuated this decrease (Fig. 3A). The changes in expression of these mRNA in each group were similar to the changes of the proteins. The mRNA levels of these proteins were similar in sham animals regardless of vehicle or chlorogenic acid treatment. Adenosylhomocysteinase levels were 0.56 ± 0.03 in vehicle + MCAO and 0.73 ± 0.04 in chlorogenic acid + MCAO. Glycerol-3-phosphate dehydrogenase levels were 0.48 ± 0.01 and 0.88 ± 0.02 in vehicle + MCAO and chlorogenic acid + MCAO animals, respectively. Eukaryotic initiation factor 4A- II levels were 0.40 ± 0.02 and 0.87 ± 0.02 in vehicle + MCAO and chlorogenic acid + MCAO animals, respectively. Apolipoprotein A-I levels were 0.55 ± 0.02 in vehicle + MCAO and 0.99 ± 0.03 in chlorogenic acid + MCAO animals. Mu-crystallin levels were 0.43 ± 0.03 in vehicle + MCAO and 0.82 ± 0.03 in chlorogenic acid + MCAO animals (Fig. 3B).

## Discussion

Ischemic brain damage causes neurobehavioral disorders and severe cerebral infarction [21]. This study demonstrated the benefits of chlorogenic acid in MCAO-induced ischemic stroke models. We previously demonstrated the neuroprotective effect of chlorogenic acid in focal cerebral ischemia by performing neurobehavioral tests and oxidative stress measurement [18]. Chlorogenic acid improves neurobehavioral disorders caused by MACO damage and reduces infarction volume. It also alleviates oxidative stress caused by MCAO [18]. Moreover, it attenuates the activation of neuroinflammatory factors and the activation of apoptotic processes in focal cerebral ischemia [18, 19]. Our supplementary results showed that chlorogenic acid mitigates the increase in DCF and MDA levels due to MCAO damage. Therefore, we clearly elucidated that chlorogenic acid exerts anti-oxidative, anti-inflammatory, and anti-apoptotic effects on MCAO-induced cerebral ischemia [18–20]. In addition to the previous studies, we identified proteins that were regulated by chlorogenic acid treatment in MCAO damage using proteomic techniques. Among identified proteins, adenosylhomocysteinase, glycerol-3-phosphate dehydrogenase, eukaryotic translation initiation factor 4A-II, apolipoprotein A-1, and mu-crystallin were the most notable. These proteins regulate various functions such as metabolism, inflammation, protein synthesis, and cell protection. Ischemic injury induces decreases in these proteins, and chlorogenic acid alleviates these decreases. These proteins are considered to be important proteins that perform the neuroprotective action of chlorogenic acid in MCAO damage. Therefore, in this study, we analyzed the expression changes of adenosylhomocysteinase, eukaryotic initiation factor 4A-II, apolipoprotein A-I, mu-crystallin, and glycerol-3-phosphate dehydrogenase.

Adenosylhomocysteinase is known as an S-adenosylhomocysteine synthase. It is found in the neocortex, hippocampus, and cerebellum [22]. S-adenosylhomocysteine hydrolase is an enzyme that catalyzes the reversible conversion of S-adenosylhomocysteine into homocysteine and adenosine [23]. The formation of S-adenosylhomocysteine occurs through transmethylation by S-adenosylmethionine, which is later broken down to homocysteine and adenosine through S-adenosylhomocysteine hydrolase [24]. Adenosine has four G protein-binding adenosine receptors, A1, A2A, A2B, and A3, among these receptors, A1 receptors are abundant in the brain [25]. The adenosine A1 receptor has a neuroprotective effect. Adenosine plays several physiological roles, including regulation of neuronal activity and release of neurotransmitters in the brain [21]. It acts as a neuroprotective agent in ischemia or hypoxia [26]. The reduction of adenosylhomocysteinase induces a decrease of adenosine and homocysteine levels. Deficiency of adenosylhomocysteinase leads to developmental brain disorders and fetal hydrops in humans [27]. Therefore, maintenance of adenosylhomocysteine in the brain is thought to play an important role in neuroprotective functions against brain damage. We have previously reported a decrease in adenosylhomocysteine during cerebral ischemia [28]. The reduction of adenosylhomocysteine reduces the production of adenosine and homocysteine and prevents the neuroprotective effect of adenosine. In this study, we confirmed a reduction in adenosylhomocysteine in MCAO damage and also identified the mitigation effect of chlorogenic acid in this reduction caused by MCAO damage. These results show that chlorogenic acid maintains levels of adenosylhomocysteine during ischemic brain injury. Thus, our findings suggest that chlorogenic acid has neuroprotective effects by controlling the level of adenosylhomocysteinase in cerebral ischemia.

Glycerol-3-phosphate dehydrogenase is an enzyme that catalyzes the reversible redox reaction of dihydroxyacetone phosphate and NADH to l-α-glycerol 3-phosphate and NAD^+^, respectively [29]. It is expressed in neurons, astrocytes, and oligodendrocytes of the brain [30, 31]. It also plays an important role in the brain. Glycerol-3-phosphate dehydrogenases have two forms: mitochondrial glycerol-3-phosphate dehydrogenase and cytosolic glycerol-3-phosphate dehydrogenase [32]. Mitochondrial glycerol-3-phosphate dehydrogenase is an essential part of the mammalian respiratory chain and glycerophosphate shuttle, serving as a bridge between mitochondria and cytoplasmic processes [33]. Cytosolic glycerol-3-phosphate dehydrogenase reduces dihydroxyacetone phosphate to glycerol-3-phosphate in the presence of NADH and H+ [31, 34]. The disruption of glycerol-3-phosphate dehydrogenase by NADH oxidase overexpression causes growth defects and reduces glycerol production [35]. Glycerol acts as a neuroprotector against neuronal damage caused by cerebral ischemia and reduces adherence of leukocytes that can interfere with blood cells and plasma flow [36]. Chlorogenic acid has the ability to regulate glycerol production [37]. In this study, our results clearly showed a significant decrease in the expression level of glycerol-3-phosphate dehydrogenase in the MCAO animals with vehicle treatment. Chlorogenic acid alleviates this decrease. These results have been identified using proteomics and reverse transcription PCR techniques. Ischemia can also reduce the mitochondrial capacity for respiratory activity and contribute to energy metabolism disorders, causing pathophysiological changes [38]. Glycerol-3-phosphate dehydrogenase plays an important role in the regulation of energy metabolism and the survival of neurons. The maintenance of glycerol-3-phosphate dehydrogenase by chlorogenic acid treatment is considered to be important for protecting neurons from ischemic damage. Therefore, these results provide evidence that chlorogenic acid has neuroprotective effects on ischemia by preventing the decrease of glycerol-3-phosphate dehydrogenase.

Translation of mRNA is a complex process that proceeds through four main phases of initiation, elongation, termination, and ribosome recycling. Among these phases, the initiation step is the most important and rate-limiting step of protein synthesis [39]. Eukaryotic translation initiation factors are the main regulators of this initiation stage that stabilize the formation of functional ribosome complexes in the starting codon [40]. Eukaryotic translation initiation factors play an important role in attaching mRNA to the 43S pre-initiation complex to initiate the protein synthesis process [41]. Eukaryotic translation initiation factor 4A belongs to the group of DEAD-box proteins that release RNA [42]. It also has helicase activity and regulates the rate of translation initiation for various mRNAs. It has three types of genes, including eukaryotic translation initiation factors 4A-I, 4A-II, and 4A-III [43]. Eukaryotic translation initiation factors 4A-I and 4A-II participate in initiating the translation process [44]. The regulation of translation initiation and elongation plays an important role in learning and memory function. However, abnormal translation initiation processes can cause a variety of cognitive disorders [45]. Eukaryotic translation initiation factor 4A-II is very important for the combination of mRNA and ribosomes and plays an important role in the initiation of translation [46]. We identified a decrease in the expression of eukaryotic translation initiation factor 4A-II in MCAO-damaged animals. The decrease in this protein in the brain represents neuronal cell damage because eukaryotic translation initiation factor 4A-II contributes to important factors in protein synthesis. In this study, we showed that chlorogenic acid prevents the reduction of MCAO-induced eukaryotic translation initiation factor 4A-II. A proteomic study and reverse transcription PCR techniques confirmed these changes. Maintenance of eukaryotic translation initiation factor 4A-II is an important event that performs normal protein synthesis. Thus, these results provide evidence that chlorogenic acid normally initiates translation and performs protein synthesis by mitigating the reduction of eukaryotic translation initiation factor 4A-II during cerebral ischemia, thereby preventing neuronal cell death from ischemic damage.

Lipoproteins are spherical macromolecules involved in the transport of triglyceride and cholesterol. They have hydrophobic and hydrophilic coatings consisting of phospholipids, cholesterol esters, free cholesterol, and apolipoprotein molecules [47]. Apolipoprotein A-I is a 28 kDa protein that is found in high-density lipoprotein (HDL) and chylomicrons [48]. Apolipoprotein A-I is 70% of all plasma HDLs and the outflow of HDL and apolipoprotein A-I can change lipid structure and reduce neutrophil activation and migration, which means that it indirectly prevents neutrophil-induced inflammation [49]. In endothelial cells, apolipoprotein A-I also prevents inflammation by increasing the level of anexin A1 and blocking the stimulation of phospholipase A2 [50]. Apolipoprotein A-I reverses the cholesterol transport pathway of the lymphatic system and prevents arteriosclerosis, thereby exhibiting vascular protection [51]. In addition, apolipoprotein A-I and HDL prevent the progression of neurodegenerative diseases such as Alzheimer's disease because they reduce β-amyloid plaques and prevent inflammation caused by β-amyloid in the brain [52]. Moreover, apolipoprotein A-I has an antioxidant effect and its overexpression reduces neuroinflammation and improves memory deficit in Alzheimer’s disease models [53]. Chlorogenic acid improves cognitive function and increases level of apolipoprotein A-I in blood [54]. We have previously reported a decrease in apolipoprotein A-I in cerebral ischemia animal models [55]. In this study, we confirmed the significant reduction of apolipoprotein A-I in MCAO damage and further explained the alleviation of this reduction by treatment with chlorogenic acid. These results showed that chlorogenic acid regulates apolipoprotein A-I expression in cerebral ischemia. These findings suggest that maintenance of apolipoprotein A-I can protect brain tissue from neuroinflammatory and neurodegenerative diseases.

Mu-crystallin is a cytoplasmic protein first discovered as a major structural component of eye lenses in Australian marsupials [56]. However, it has been found in many other mammals such as humans and mice and is known to exist in the eyes, ears, heart, and brain [57]. In the brain, it is expressed highly in the cerebral cortex and hippocampus [58]. Mu-crystallin is known as the NADPH-dependent cytoplasm 3,5,3-triiodo-l-thyronine thyroid-hormone-binding protein and can also act as a ketamine reductase [59]. Thyroid hormones are the major regulatory factors for gene expression and metabolism. Mu-crystallin plays an essential role in regulating thyroid hormone levels that contribute to physiological metabolism [60]. In addition, mu-crystallin level decreases in the caudate nucleus and cerebellum of patients with Huntington’s disease. A decrease in mu-crystallin level in the dorsal prefrontal cortex can also be found in patients suffering from schizophrenia [61]. These reductions in neurological diseases mean that mu-crystallin plays an important role in brain disease [62]. Mu-crystallin plays a key role in maintaining normal physiological metabolism and improving the plasticity of the brain. This study confirms a decrease in mu-crystallin expression in the cerebral cortex of MCAO damaged animal. Previous studies have demonstrated that reduction of mu-crystallin leads to neurological disorders [60–62]. Our results show that reduction of mu-crystallin due to MCAO damage is improved in chlorogenic acid-treated animals. Thus, our findings demonstrate the regulation of mu-crystallin by chlorogenic acid treatment in ischemia damage. These results have been confirmed by proteomic and the reverse transcription PCR technique. More research is needed to explain the mechanism of regulation of mu-crystallin by chlorogenic acid. However, we demonstrated that mitigation of mu-crystallin reduction by chlorogenic acid treatment in cerebral ischemia contributes to the neuroprotective effect of chlorogenic acid.

In summary, chlorogenic acid prevents the reduction of adenosylhomocysteinase, glycerol-3-phosphate dehydrogenase, eukaryotic translation initiation factor 4A-II, apolipoprotein A-1, and mu-crystallin due to MCAO damage. Adenosylhomocysteinase and glycerol-3-phosphate dehydrogenase are catalytic enzymes that regulate metabolism. Eukaryotic translation initiation factor 4A-II contributes to protein synthesis, and apolipoprotein A-1 regulates lipid homeostasis and inflammation. Mu-crystallin is involved in physiological metabolism. The proteins mentioned in the discussion are related to neuroprotection against brain damage.

## Conclusions

Our findings demonstrate that chlorogenic acid performs a neuroprotective effect against cerebral ischemia through the regulation of several proteins. This study also provides new information on the protective mechanisms of chlorogenic acid in ischemic animal models.

## Methods

### Experimental animals and drug treatment

Male Sprague Dawley rats (*n* = 32, 7 weeks age, 200–220 g) were used in experimental procedures and were purchased from Samtako Bio Korea (Osan, Korea). All experimental procedures were performed according to the approved guidelines of the Institutional Animal Care and Use Committee of Gyeongsang National University (GNU-220222-20021). Animals were kept in an animal room with a controlled temperature (25 °C) and lightning (12 h light/12 h dark cycle) system for 1 week before drug treatment for acclimatization to the new environment. Feed and water were supplied freely to animals. Animals were randomly divided into four groups: vehicle + sham, chlorogenic acid + sham, vehicle + middle cerebral artery occlusion (MCAO), and chlorogenic acid + MCAO group. Phosphate buffer saline (PBS) was used as a vehicle and chlorogenic acid (Sigma-Aldrich, Saint-Louis, MO, USA) was dissolved in PBS. Chlorogenic acid (30 mg/ kg) or vehicle was injected intraperitoneally 2 h before MCAO procedure [10].

### Middle cerebral artery occlusion

MCAO was carried out as a previously reported procedure [63]. Zoletil (50 mg/kg, Virbac, Carros, France) was injected intramuscularly for induction of anesthesia in all animals. Animals were carefully placed on heating pads to prevent hypothermia in a supine position. A midline incision was made in the neck and right common carotid artery (CCA) was exposed. External carotid artery (ECA) and internal carotid artery (ICA) were properly exposed and separated from nearby tissues. The end of supra thyroid artery and occipital artery were permanently ligated and cut in the middle. The right CCA was temporarily blocked with a microvascular clamp and proximal end of the ECA was ligated. ECA was cut and nylon filament (4-0) with blunted tip by heating was inserted into ECA. We pushed nylon into ECA until the resistance was felt to block the origin of middle cerebral artery. The length of inserted nylon filament was almost 22–24 mm. Nylon suture was ligated at the proximal end of the ECA with a silk suture to fix the nylon suture. Incision line was closed with a black suture and animals were kept on heating pad until they woke up from anesthesia. They were moved to animal cages and neurological behavior tests were carried out after 24 h of MCAO.

### Reactive oxygen species assay

The ROS assay was performed using a ROS assay kit (Elabscience^©^ Biotechnology Inc. Houston, Texas, USA) according to the manual provided by the manufacturer. We measured the amount of ROS by single cell suspension method. The isolated right cerebral cortex tissue was dissolved with a lysis buffer. The dissolved tissues were mixed with a reagent 3 working solution and then reacted with an enzyme solution to digest the tissue at 37 °C for 30 min. The digestion process was stopped by adding the reagent 3 working solution to the mixture. The mixture was filtered, centrifuged at 1000× *g* and the supernatant was discarded. The obtained cortical cells were mixed with the reagent 1 working solution. Cells were reacted with 5 mM 2′, 7′-dichlorodihydrofluorescein diacetate (DCFH-DA) at 37 °C for 1 h. This process leads to the conversion of DCFH-DA into the fluorescent product DCF. After incubation, the cells were washed with reagent 3 working solution and centrifuged at 1000× *g* for 10 min. The precipitated cells were scanned with Multiskan EX spectrophotometer (Thermo Fisher Scientific, Waltham, MA, USA) at an excitation wavelength of 500 nm and an emission wavelength of 525 nm. The obtained values were expressed as tissue relative DCF pmol/mg.

### Lipid peroxidation assay

The LPO assay was performed to measure the level of oxidative stress through MDA, an LPO biomarker. It was performed using a LPO assay kit (Biovision Incorporated, Milpitas, CA, USA) according to the manual provided by the manufacturer. The right cerebral cortex tissue was isolated and dissolved in lysis buffer contained with butylated hydroxytoluene. The resulting mixture was centrifuged at 13,000× *g* for 10 min. The supernatant was collected and reacted with thiobarbituric acid. The mixture was incubated at 95 °C for 1 h and kept on ice, and the absorbance was measured at 532 nm using Multiskan EX spectrophotometer (Thermo Fisher Scientific, Waltham, MA, USA). The results were expressed as nmol/mg protein.

### Two-dimensional gel electrophoresis

Right cerebral cortex tissues were homogenized in lysis buffer (8 M urea, 4% CHAPS, 0.2% Bio-Lyte, 40 mM Tris–Hcl) for the preparation of protein samples. The homogenates were centrifuged at 15,000 g for 30 min at 4 °C and the supernatants were collected and precipitated in 10% trichloroacetic acid for 30 min at room temperature. They were centrifuged at 14,000 g for 15 min at 4 °C for condensation of protein and the supernatants were discarded. The pellets were washed with 1 M Tris–Hcl (pH 7.6) and dried at room temperature. They were mixed with sample buffer [8 M urea, 4% CHAPS, 0.2% Bio-Lyte, 40 mM Tris–Hcl, 1% (v/v) pharmalytes, 2 µg/ml dithiothreitol (DTT)] and the mixtures were sonicated for 3 min, and then kept for 1 h at room temperature. The mixture was centrifuged and supernatant was collected from each sample. Protein concentration was measured with Bradford protein assay kit (Bio-Rad, Hercules, CA, USA). For first dimensional isoelectric focusing, total protein (50 µg) were mixed with rehydrating solution (8 M urea, 2% CHAPS, 20 mM DTT, 0.5% IPG buffer, bromophenol blue) room temperature overnight and the mixtures were loaded to immobilized pH gradient (IPG) gel strips (Immobiline DryStrip, pH 4–7, 17 cm, Bio-Rad). Isoelectric focusing was performed using an Ettan IPGphor 3(GE Healthcare, Uppasala, Sweden) with following conditions: voltage: 200 V (1 h), 500 V (1 h), 1000 V (1 h), 8000 V (30 min), and 8000 V (5–6 h). For second dimensional electrophoresis, IPG strips were reacted with equilibration buffer [6 M urea, 30% glycerol, 2% sodium dodecyl sulfate (SDS), 50 mM Tris–Hcl, bromophenol blue] with 1% DTT for 10 min, and incubated with equilibration buffer containing 2.4% iodoacetamide for 10 min. They were loaded into gradient gels (7.5–17.5%), and 2D gel electrophoresis (2-DE) was performed with Protein-II XI electrophoreses equipment (Bio-Rad) at 15 °C, 10 mA until the blue dye went down to the bottom of the gel. The gels were carefully removed from the glass plate and kept in a fixing solution (12% acetic acid in 50% methanol) for 2 h at room temperature. They were washed twice with 50% ethanol for 20 min, reacted with sodium thiosulphate for 10 min to sensitize the gels, and washed with distilled water. The gels were stained with a silver stain solution (0.2% silver nitrate, 0.75 ml/L 37% formaldehyde) for 20 min, and washed with distilled water two times for 1 min. Stained gels were developed in a developing solution (2% sodium carbonate, 0.5 ml/L 37% formaldehyde) for approximately 5 min until each protein spot on the gel was clearly visible. They were reacted with a stop solution (1% acetic acid) for 15 min to stop developing step. The gels were scanned with Agfar ARCUS 1200™ (Agfar-Gevaert, Mortsel, Belgium), and images of gels were taken. Images were analyzed using PDQuest 2-DE analysis software and proteins with differential expression among groups were identified. For matrix-assisted laser desorption/ionization-time of flight (MALDI-TOF) analysis, protein spots from gels were cut and reacted with destained solution (30 mM potassium hexacyanoferrate, 100 mM sodium thiosulfate). Protein spots were washed with a washing solution (10% acetic acid in 50% methanol) to remove silver stain solution. They were dehydrated with 50 mM of ammonium bicarbonate and acetonitrile, dried in a vacuum centrifuge, and then incubated in a reduction solution (10 mM DTT in 0.1 M ammonium bicarbonate) at 56 °C for 45 min. Gel spots were dehydrated in 0.1 M ammonium bicarbonate and acetonitrile, and dried in a vacuum centrifuge for 20 min. Dried spots were digested in a digestion solution (12.5 ng/ml trypsin, 0.1% octyl beta-D glycopyranside in 50 mM ammonium bicarbonate) at 37 °C overnight and reacted with an extraction buffer (1% trifluoroacetic acid in 66% acetonitrile) to collect trypsin digested proteins. The extracted proteins were centrifuged in a vacuum centrifuge for 2 h and dried. For the identification of specific proteins, dried proteins were melted in nitrocellulose mixed in alpha-cyano-4-hyroxycinnamic acid (CHCA) in acetone. The resultant solutions were mixed up in a ratio of 1:4 and the final matrix solution was made with calibrants. The extracted proteins were mixed with extraction buffer by pipetting and loaded to a MALDI-TOF plate. After drying on MALDI-TOF plate, MALDI-TOF was performed using a Voyager System-DE-STR mass spectrometer (Applied Biosystem, Foster City, CA, USA). Analysis of the peak results was done online on NCBI and MS-FIT websites.

### Reverse transcription polymerase chain reaction

Right cerebral cortices were dissolved in Trizol Reagent (Life Technologies, Rockville, MD, USA) for extraction of total RNA as instructed method by the manufacturers. Total RNA (1 µg) was used for the synthesis of single-stranded complementary DNA with the Superscript III first-strand system (Invitrogen, Carlsbad, CA, USA) as the manual of manufacturer. Amplification of the targeted genes was performed by polymerase chain reaction (PCR) reaction with manufactured specific primers. The primer sequences used in this experiment was summarized in Table 2. PCR reaction was carried out as following steps: denaturation for 5 min at 94 °C; 30 cycles of denaturation step at 94 °C for 30 s, annealing step at 54 °C for 30 s, and elongation step at 72 °C for 1 min; and a final extension for 10 min at 72 °C. The PCR product was electrophoresed in 1% agarose gel and detected under an ultraviolet light. Images were captured and the intensity of PCR bands was measured with Image J computer-based software (National Institute of Health, Bethesda, MD, USA). The expression level of specific genes was expressed as a ratio of PCR product density to β-actin density.

**Table 2:** List of forward and reverse primers used for amplification

Gene	Primer sequences (F, Forward; R, Reverse)	Product (bp)
Adenosylhomocysteinase	F: 5′-AAGCTGCCATGGAGGGCTAC3′ R: 5′-GATGGCAGCTGGAAGGTGAA-3′	583
Glycerol-3-phosphate dehydrogenase	F: 5′-GGCCCTTTCACAGACTCCGT-3′ R: 5′-TCCATGTTCTCGGGGCTGT-3′	117
Eukaryotic initiation factor 4A-II	F: 5′-CAGAGGGAATGGACCCCGAT-3′ R: 5′-GTGGCTGTCTTGCCAGTACC-3′	214
Apolipoprotein A-I	F: 5′-GGGAGTTCTGGCAGCAAGAT-3′ R: 5′-GCTGTTTGCCCAAAGTGGAG-3′	138
Mu-crystallin	F: 5′-TCCCAGCAATGGTTCCCTGC-3′ R: 5′-TATCACTGCCTGGGGGCTTC-3′	113
β-actin	F: 5′-TACAACCTTCTTGCAGCTCCTC-3′ R: 5′-CCTTCTGACCCATACCCACC-3′	205

### Statistical analysis

All results are represented as mean ± standard error of means (S.E.M.). Data from all groups were analyzed by two-way analysis of variance (ANOVA) followed by *post-hoc* Scheffe’s test. A p-value of less than 0.05 (*p* < 0.05) was considered to be statistically significant.

## Supplementary Information

**Additional file 1.** Chlorogenic acid attenuates the increase in oxidative stress due to MCAO damage. Reactive oxygen species and lipid peroxidation analyses in the cerebral cortex from vehicle + sham, chlorogenic acid + sham, vehicle + middle cerebral artery occlusion, CGA + MCAO animals. MCAO damage increased 2′7′‐dichlorofluoresceinand malondialdehydelevels, chlorogenic acid attenuates these increases. Dataare represented as the mean ± S.E.M. #*p* < 0.01 versus vehicle + sham animals, *p < 0.05 versus vehicle + MCAO animals.

## Data Availability

The data that support the findings of this study are available on request from the corresponding author on reasonable request.

## Abbreviations

CCA: Common carotid artery
CGA: Chlorogenic acid
CHAPS: 3-[(3-Cholamidopropyl)dimethylammonio]-1-propanesulfonate.
CHCA: Alpha-cyano-4-hyroxycinnamic acid
DCF: 2′7′‐Dichlorofluorescein
DTT: Dichlorodiphenyltrichloroethane
ECA: External carotid artery
ICA: Internal carotid artery
IPG: Immobilized pH-gradient
LPO: Lipid peroxidation
MCAO: Middle cerebral artery occlusion
MALDI-TOF: Matrix-assisted laser desorption/ionization-time of flight
MDA: Malondialdehyde
PBS: Phosphate-buffered saline
PCR: Polymerase chain reaction
ROS: Reactive oxygen species
SDS: Sodium dodecyl sulphate
2-DE: Two-dimensional gel electrophoresis
LPO: Lipid peroxidation
MDA: Malondialdehyde
