# Supplementary figures and images for: Identification of proteins regulated by chlorogenic acid in an ischemic animal model: a proteomic approach
Source: Lab Anim Res. 2023 Jun 5;39:12. doi: 10.1186/s42826-023-00164-5 (PMC10240784; doi:10.1186/s42826-023-00164-5)

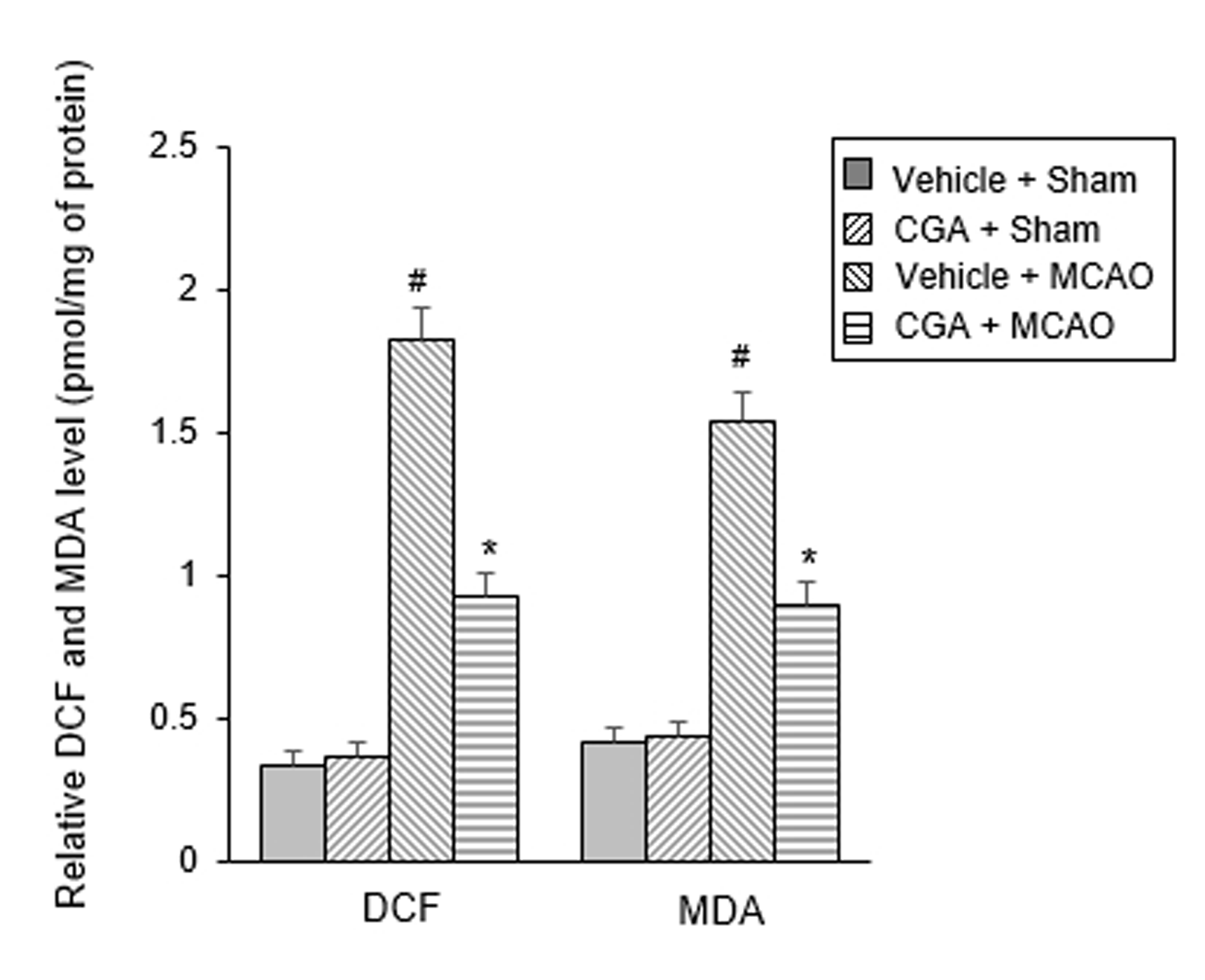

Supplement: Supplementary file 1 — Additional file 1. Chlorogenic acid attenuates the increase in oxidative stress due to MCAO damage. Reactive oxygen species and lipid peroxidation analyses in the cerebral cortex from vehicle + sham, chlorogenic acid + sham, vehicle + middle cerebral artery occlusion, CGA + MCAO animals. MCAO damage increased 2′7′‐dichlorofluoresceinand malondialdehydelevels, chlorogenic acid attenuates these increases. Dataare represented as the mean ± S.E.M. #p < 0.01 versus vehicle + sham animals, *p < 0.05 versus vehicle + MCAO animals. [file 42826_2023_164_MOESM1_ESM.tif]
